# A Systems Biology Approach to the Coordination of Defensive and Offensive Molecular Mechanisms in the Innate and Adaptive Host–Pathogen Interaction Networks

**DOI:** 10.1371/journal.pone.0149303

**Published:** 2016-02-16

**Authors:** Chia-Chou Wu, Bor-Sen Chen

**Affiliations:** Automatic control, Signal processing, and Systems Biology Lab, National Tsing Hua University, Hsinchu, 30013, Taiwan; King’s College London Dental Institute, UNITED KINGDOM

## Abstract

Infected zebrafish coordinates defensive and offensive molecular mechanisms in response to *Candida albicans* infections, and invasive *C. albicans* coordinates corresponding molecular mechanisms to interact with the host. However, knowledge of the ensuing infection-activated signaling networks in both host and pathogen and their interspecific crosstalk during the innate and adaptive phases of the infection processes remains incomplete. In the present study, dynamic network modeling, protein interaction databases, and dual transcriptome data from zebrafish and *C. albicans* during infection were used to infer infection-activated host–pathogen dynamic interaction networks. The consideration of host–pathogen dynamic interaction systems as innate and adaptive loops and subsequent comparisons of inferred innate and adaptive networks indicated previously unrecognized crosstalk between known pathways and suggested roles of immunological memory in the coordination of host defensive and offensive molecular mechanisms to achieve specific and powerful defense against pathogens. Moreover, pathogens enhance intraspecific crosstalk and abrogate host apoptosis to accommodate enhanced host defense mechanisms during the adaptive phase. Accordingly, links between physiological phenomena and changes in the coordination of defensive and offensive molecular mechanisms highlight the importance of host–pathogen molecular interaction networks, and consequent inferences of the host–pathogen relationship could be translated into biomedical applications.

## Introduction

The importance of host–pathogen interactions (HPIs) was recently highlighted in the infection process [1–4]. However, the gap between infection-activated molecular mechanisms and physiological phenomena restricts the translation of the knowledge from HPIs to biomedical applications [5, 6]. Hence, we used dual transcriptome data to simultaneously record the temporal gene expression profiles of the host (zebrafish) and pathogen (*Candida albicans*) during innate and adaptive phases of infection. These experiments allowed the analysis of the coordination of host and pathogen defensive and offensive molecular mechanisms in both phases. Specifically, dynamic host–pathogen protein–protein interaction networks (HP-PPINs) can be used to bridge the gap between infection-activated molecular mechanisms and physiological phenomena. Moreover, dynamic PPINs quantitatively delineate the effects of current protein levels on the expression of other proteins [7] and can, therefore, be used to characterize the molecular mechanisms behind the interactions of host and pathogen proteins during the infection process. Hence, relating the infection-activated molecular mechanisms to physiological phenomena using dynamic HP-PPINs may inform biomedical applications from the investigations of HPIs.

The infection process has been described as a battle or tug of war between host and pathogen [8, 9]. From the host perspective, innate and adaptive immunity are sequentially activated from pathogen exposure to disease recovery and correspond with the two major phases of the battle. Initially, innate immunity mediates the first line of host defensive molecular mechanisms, including pathogen recognition via the actions of several cell types including macrophages, dendritic cells, and natural killer cells, which are recruited to the sites of infection to eliminate pathogens. The recognition of pathogen-associated molecular patterns and/or damage-associated molecular patterns by pattern recognition receptors (PRRs) such as toll-like receptors and C-type lectin receptors can be viewed as an origin of the following complex molecular events [10, 11]. PRRs initiate downstream signaling pathways that activate the innate immune system to clear pathogens through the production and secretion of cytokines, chemokines, and chemotactic cues that recruit more leukocytes [12]. Subsequently, macrophages and dendritic cells process and present antigens to T cells and induce the adaptive phase. During this phase, B and T cells specifically and efficiently eliminate pathogens by producing antibodies and inducing specific types of cells to attack pathogens [13]. Considering these distinct molecular interactions with the pathogens during the two phases of the infection process, pathogens likely respond with two corresponding molecular strategies, although these remain poorly characterized.

From the pathogen perspective, the molecular mechanisms involved in resource acquisition and utilization for offensive functions are much clearer in *C. albicans* than in zebrafish. However, both pathogens and hosts require resources to support vital functions, leading to competition under the conditions of resource limitation in infected hosts. Among such resources, iron is an essential nutrient for pathogenic microbes and plays critical roles in multiple cellular processes [14]. Accordingly, *C. albicans* have strategies for acquiring iron from specific host molecules, leading to virulence and diseases [15]. Glucose also plays central roles as a carbon and energy source and as a morphogen that affects the yeast-to-hyphae transition, which is a critical determinant of optimal virulence in the host [16–20]. The mechanisms of iron and glucose competition in pathogens have been qualitatively analyzed in terms of iron-mediated gene expression [21, 22]. However, in the present study, dual transcriptome data and a dynamic interaction model enabled the quantitative descriptions of molecular mechanisms associated with pathogen resource competition and interspecific crosstalk with host counterparts during innate and adaptive phases. Moreover, corresponding host responses to pathogen resource competition, which are less studied, were further identified and analyzed based on the present innate and adaptive HP-PPINs construction.

**Fig 1:**
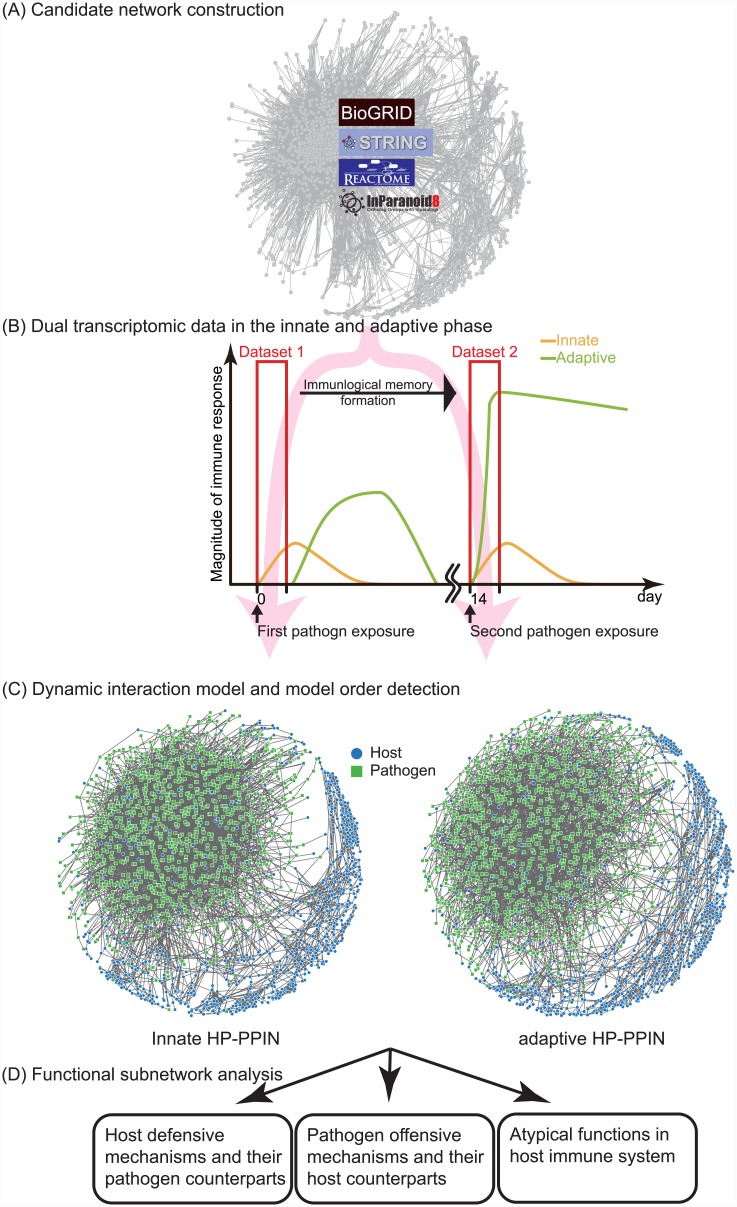
Overview of the dynamic host–pathogen protein–protein interaction network (HP-PPIN) construction and analysis procedure. (A) The Zebrafish–*C. albicans* candidate PPI network was constructed based on PPI information from BioGRID, STRING, and REACTOME databases and ortholog information from the InParanoid database. (B and C) Dual transcriptome data from innate and adaptive phases were used to prune the candidate network, identify interaction strengths between proteins in the dynamic interaction model, and to obtain dynamic innate and adaptive HP-PPINs, respectively. Blue and green nodes represent host and pathogen proteins, respectively. (D) Functional enrichment analyses revealed defensive, offensive, and atypical functions in host and pathogen.

Defenses and offenses of host and pathogen are typical study objects of infectious diseases [23, 24], whereas the coordination of defenses and offenses in each phase with other atypical molecular mechanisms remain poorly characterized. Thus, after identifying the defensive and offensive molecular mechanisms of the host and pathogen during the innate and adaptive phases, the crosstalk between these molecular mechanisms can be further investigated by observing and comparing the interaction strengths between proteins and functions in HP-PPINs. In the innate pathogen exposure phase, host molecular mechanisms lack specificity for the pathogen, in this case *C. albicans*. However, immunological memory of the initial pathogen challenge modulates subsequent specific host molecular mechanisms, although the ensuing coordination of host molecular mechanisms has not been elucidated. Hence, in the present study, we proposed a general method for the quantitative analysis of interaction strengths in the constructed networks, examined the defensive and offensive molecular mechanisms of the host and pathogen during the innate and adaptive phases, and investigated interspecific and intraspecific crosstalk (Fig 1). Subsequently, host subnetworks adjacent to HPIs indicated that neuroimmune molecular mechanisms may also be modulated by immunological memory. Hence, the comparisons between inferred networks in both phases allowed the division of host–pathogen dynamic interaction systems into innate and adaptive loops. These observations of crosstalk between known pathways indicated the mechanisms by which immunological memory in the adaptive loop coordinates host molecular mechanisms to achieve specific defense against the pathogen. In particular, pathogens enhance intraspecific crosstalk and abrogate host apoptosis to cope with enhanced host defensive mechanisms during adaptive phase. These observations and host–pathogen dynamic interaction systems may form the basis for future biomedical applications.

## Materials and Methods

### Transcriptome datasets

Transcriptome datasets included simultaneously recorded temporal expression profiles of zebrafish and *C. albicans* during the innate phase immediately after pathogen exposure (GSE32119 [25, 26]) and simultaneously recorded temporal expression profiles of zebrafish and *C. albicans* during the adaptive response to secondary pathogen exposure (GSE51603 [22]). Details of experimental procedures are described in a previous study [4]. In the first dataset, *C. albicans* (SC5314 strain) was intraperitoneally injected into adult AB-strain zebrafish (first exposure), and microarray experiments were performed to simultaneously profile genome-wide expression in *C. albicans* and zebrafish during the innate phase of the infection process. The second dataset included genome-wide expression data for *C. albicans* and zebrafish during the adaptive phase after the secondary exposure (14 days after the first exposure) to *C. albicans*. Subsequently, a two-step homogenized mRNA extraction procedure was performed using the whole *C. albicans*-infected zebrafish. This approach can provide separate pools of gene transcripts from hosts and pathogens, and the individual estimates of corresponding specific gene expression profiles of sequence-targeted probes are derived from individual genomes. Agilent *in situ* oligonucleotide microarrays cover 6,202 and 26,206 genes for *C. albicans* and zebrafish, respectively, and were used to record temporal gene expression. The first dataset comprised three replicates of host and pathogen gene expression data from 0.5, 1, 2, 4, 6, 8, 12, 16, and 18 h post-injection, and the second dataset comprised two replicates of host and pathogen gene profiles from 2, 6, 12, 18, 24, 30, 36, and 42 h post-re-injection.

### Dynamic interaction model

To construct a dynamic network, a candidate network including PPIs was gathered from several PPI databases (BioGRID [27], STRING [28], and REACTOME [29]), and false-positive interactions were then eliminated to obtain the resulting networks based on temporal expression profiles, the dynamic interaction model, and a model order detection method.

#### Candidate network

To construct a candidate network, interaction information for zebrafish–zebrafish, *C. albicans*–*C. albicans*, and zebrafish–*C. albicans* protein pairs would be ideal. However, incomplete information for all three interaction types results in incomplete networks. In addition, it is impossible to consider all interactions between proteins due to computational complexity. Thus, interaction information from human and yeast (*S. cerevisiae*), which bear genetic similarities with zebrafish and *C. albicans* were used to fill information gaps. To infer candidate interactions of zebrafish and *C. albicans* orthologous information from Inparanoid [30] was used to convert the interactions of human and yeast proteins into those of zebrafish and *C. albicans* proteins (Fig 1A). Among these, 1,216 pathogen proteins and 1,087 host proteins were included in the candidate network, comprising 5,347 host–host interactions, 3,634 host–pathogen interactions, and 16,622 pathogen–pathogen interactions. Interactions collected from databases and inferred by ortholog-based methods were derived under various experimental conditions, and may not be present during the *C. albicans* infection process. Accordingly, false-positive interactions were validated and removed using experimental data and the model order detection method (Fig 1B). That is, to reduce false-positive PPI information in the candidate network, system identification techniques were used with microarray data. After deleting false-positive interactions from the candidate network, dynamic HP-PPINs in innate and adaptive phases were then generated using dual transcriptome data and the following dynamic interaction model.

#### Dynamic interaction model and model order detection method

Total numbers of host and pathogen proteins were denoted as *N* and *M*, respectively, and the dynamic interaction model of a host protein *i* in the HP-PPIN is described as follows [31]:

$$\begin{array}{ll} p_{i}^{\left(h\right)}\left[k+1\right] & =\sigma_{i}^{\left(h\right)}p_{i}^{\left(h\right)}\left[k\right]+\sum_{n=1}^{N}\alpha_{in}^{\left(h\right)}p_{n}^{\left(h\right)}\left[k\right]p_{i}^{\left(h\right)}\left[k\right]\\ & \;+\sum_{m=1}^{M}\gamma_{im}p_{m}^{\left(p\right)}\left[k\right]p_{i}^{\left(h\right)}\left[k\right]+\beta_{i}^{\left(h\right)}+\epsilon_{i}^{\left(h\right)}\left[k+1\right] \end{array}$$ (1)

where $p_{i}^{\left(h\right)}\left[k\right]$ denotes the expression of host protein *i* at time *k*, $\sigma_{i}^{\left(h\right)}\left[k\right]$ denotes the environmental noise at time *k*, $\sigma_{i}^{\left(h\right)}$ denotes the transition ability of the current (at time *k*) to the future (at time *k*+1) expression level of the host protein *i*, and $\alpha_{in}^{\left(h\right)}$ denotes the interaction strength between host proteins *n* and *i*. Hence, if there is no interaction between host protein *n* and *i*, $\alpha_{in}^{\left(h\right)}=0$. We also assumed there is no self-interaction of protein *i* ($\alpha_{ii}^{\left(h\right)}=0$). *γ*_*im*_ denotes the interaction strength between pathogen protein *m* and host protein *i*. If there is no interaction between pathogen protein *m* and host protein *i*, *γ*_*im*_ = 0. $\beta_{i}^{\left(h\right)}$ denotes the basal level of the host protein *i*, which is greater than or equal to 0. The term “transition ability” is coined to evaluate the impact of current protein levels ($p_{i}^{\left(h\right)}\left[k\right]$) on future protein levels ($p_{i}^{\left(h\right)}\left[k+1\right]$). In the biological sense, multiple factors, such as protein degradation rates, can affect the transition abilities, and the dynamic interaction model of a pathogen protein *j* in the HP-PPIN can be written as follows:

$$\begin{array}{ll} p_{j}^{\left(p\right)}\left[k+1\right] & =\sigma_{j}^{\left(p\right)}p_{j}^{\left(p\right)}\left[k\right]+\sum_{m=1}^{M}\alpha_{jm}^{\left(p\right)}p_{m}^{\left(p\right)}\left[k\right]p_{j}^{\left(p\right)}\left[k\right]\\ & \;+\sum_{n=1}^{N}\gamma_{jn}p_{n}^{\left(h\right)}\left[k\right]p_{j}^{\left(p\right)}\left[k\right]+\beta_{j}^{\left(p\right)}+\epsilon_{j}^{\left(p\right)}\left[k+1\right] \end{array}$$ (2)

where $p_{j}^{\left(p\right)}\left[k\right]$ denotes the expression of the pathogen protein *j* at time *k*, $\sigma_{j}^{\left(p\right)}\left[k\right]$ denotes the environmental noise at time *k*, $\sigma_{j}^{\left(p\right)}$ denotes the transition ability of the current (at time *k*) to the future (at time *k*+1) protein level of the pathogen protein *j*, $\alpha_{jm}^{\left(p\right)}$ denotes the interaction strength between pathogen proteins *m* and *j* (no interaction between pathogen protein *m* and pathogen protein *j*, $\alpha_{jm}^{\left(p\right)}=0$; $\alpha_{jj}^{\left(p\right)}=0$), *γ*_*jn*_ denotes the interaction strength between host protein *n* and pathogen protein *j* (no interaction between host protein *n* and pathogen protein *j*, *γ*_*jn*_ = 0), and $\beta_{j}^{\left(p\right)}$ denotes the basal pathogen protein *j* expression, which is greater than or equal to 0. The biological relevance of the dynamic interaction model follows the determination of the host (pathogen) protein *i* (*j*) expression in the future (at time *k*+1) according to current (at time *k*) protein expression with transition ability $\sigma_{i}^{\left(h\right)}$ ($\sigma_{j}^{\left(p\right)}$), the interaction between host (pathogen) protein *i* (*j*) and other host (pathogen) proteins with interaction strengths $\alpha_{in}^{\left(h\right)}$ ($\alpha_{jm}^{\left(p\right)}$), the interaction between host (pathogen) protein *i* (*j*) and other pathogen (host) proteins with interaction strengths *γ*_*im*_ (*γ*_*jn*_), its basal level $\beta_{i}^{\left(h\right)}$ ($\beta_{j}^{\left(p\right)}$), and the environmental noise $\sigma_{i}^{\left(h\right)}$ ($\sigma_{j}^{\left(p\right)}$). Hence, the dynamic interaction model for host protein *i* of *K*+1 time points (*k* = 1,⋯, *K*+1) can be further rewritten as follows:

$$p_{i}^{\left(h\right)}=\Phi_{i}^{\left(h\right)}\theta_{i}^{\left(h\right)}+\epsilon_{i}^{\left(h\right)}$$ (3)

where $p_{i}^{\left(h\right)}={\left[p_{i}^{\left(h\right)}\left[2\right]\;\cdots \;p_{i}^{\left(h\right)}\left[K+1\right]\right]}^{T}$, $\epsilon_{i}^{\left(h\right)}={\left[\epsilon_{i}^{\left(h\right)}\left[2\right]\;\cdots \;\epsilon_{i}^{\left(h\right)}\left[K+1\right]\right]}^{T}$, $\theta_{i}^{\left(h\right)}={\left[\alpha_{i1}^{\left(h\right)}\;\cdots \;\alpha_{iN}^{\left(h\right)}\gamma_{i1}\;\cdots \;\gamma_{iM}\sigma_{i}^{\left(h\right)}\beta_{i}^{\left(h\right)}\right]}^{T}$, and

$$\Phi_{i}^{\left(h\right)}=\left[\begin{array}{cccccccc} p_{1}^{\left(h\right)}[1]p_{i}^{\left(h\right)}[1] & \cdots & p_{N}^{\left(h\right)}[1]p_{i}^{\left(h\right)}[1] & p_{1}^{\left(p\right)}[1]p_{i}^{\left(h\right)}[1] & \cdots & p_{M}^{\left(p\right)}[1]p_{i}^{\left(h\right)}[1] & p_{i}^{\left(h\right)}[1] & 1\\ \vdots & \ddots & \vdots & \vdots & \ddots & \vdots & \vdots & \vdots \\ p_{1}^{\left(h\right)}[K]p_{i}^{\left(h\right)}[K] & \cdots & p_{N}^{\left(h\right)}[K]p_{i}^{\left(h\right)}[K] & p_{1}^{\left(p\right)}[K]p_{i}^{\left(h\right)}[K] & \cdots & p_{M}^{\left(p\right)}[K]p_{i}^{\left(h\right)}[K] & p_{i}^{\left(h\right)}[K] & 1 \end{array}\right]$$

The dynamic model for pathogen protein *j* of *K*+1 time points (*k* = 1,⋯, *K*+1) can also be rewritten into a similar form:

$$p_{j}^{\left(p\right)}=\Phi_{j}^{\left(p\right)}\theta_{j}^{\left(p\right)}+\epsilon_{j}^{\left(p\right)}$$ (4)

where $p_{j}^{\left(p\right)}={\left[p_{j}^{\left(p\right)}\left[2\right]\;\cdots \;p_{j}^{\left(p\right)}\left[K+1\right]\right]}^{T}$, $\epsilon_{j}^{\left(p\right)}={\left[\epsilon_{j}^{\left(p\right)}\left[2\right]\;\cdots \;\epsilon_{j}^{\left(p\right)}\left[K+1\right]\right]}^{T}$, $\theta_{i}^{\left(p\right)}={\left[\alpha_{j1}^{\left(p\right)}\;\cdots \;\alpha_{jN}^{\left(p\right)}\gamma_{j1}\;\cdots \;\gamma_{jN}\sigma_{j}^{\left(p\right)}\beta_{j}^{\left(p\right)}\right]}^{T}$, and

$$\Phi_{j}^{\left(p\right)}=\left[\begin{array}{cccccccc} p_{1}^{\left(p\right)}[1]p_{j}^{\left(p\right)}[1] & \cdots & p_{M}^{\left(p\right)}[1]p_{j}^{\left(p\right)}[1] & p_{1}^{\left(h\right)}[1]p_{j}^{\left(p\right)}[1] & \cdots & p_{N}^{\left(h\right)}[1]p_{j}^{\left(p\right)}[1] & p_{j}^{\left(p\right)}[1] & 1\\ \vdots & \ddots & \vdots & \vdots & \ddots & \vdots & \vdots & \vdots \\ p_{1}^{\left(p\right)}[K]p_{j}^{\left(p\right)}[K] & \cdots & p_{M}^{\left(p\right)}[K]p_{j}^{\left(p\right)}[K] & p_{1}^{\left(h\right)}[K]p_{j}^{\left(p\right)}[K] & \cdots & p_{N}^{\left(h\right)}[K]p_{j}^{\left(p\right)}[K] & p_{j}^{\left(p\right)}[K] & 1 \end{array}\right]$$

The unknown parameter $\theta_{i}^{\left(h\right)}$ ($\theta_{j}^{\left(p\right)}$) can then be estimated using the least-squares estimation method with linear constraint ($\beta_{i}^{\left(h\right)}>0$ for host; $\beta_{j}^{\left(p\right)}>0$ for pathogen). Constraint least square problems were solved using the lsqlin function in MATLAB with the active-set algorithm. The residual sum of squares for each gene would be calculated and plotted the distribution. Some examples of the comparison between measurement and estimation would be presented in the figures in S1 Fig. Because of the unavailability of host and pathogen protein expressions, gene expressions were measured using dual transcriptome data as a substitute for protein levels to estimate the parameter $\theta_{i}^{\left(h\right)}$ ($\theta_{j}^{\left(p\right)}$) in dynamic interaction models. Although protein and gene expression levels do not always correspond accurately, gene expressions are reasonably used to represent protein expressions in the current context [32]. Before estimating, expression data were interpolated using cubic spline data interpolation. To limit unnecessarily complex, Akaike information criterion (AIC) was introduced to detect model order (numbers of interactions) during the parameter estimation of dynamic HP-PPINs in Eqs (1) and (2). For each host protein *i* with *N*′ interacting host proteins and *M*′ interacting pathogen proteins, the AIC value of its dynamic interaction model can be calculated as follows [33]:

$$AIC_{i}\left(N^{\prime }+M^{\prime }\right)=\log\frac{1}{K+1}{\left\|\begin{array}{c} p_{i}^{\left(h\right)}-\Phi_{i}^{\left(h\right)}{\hat{\theta }}_{i}^{\left(h\right)} \end{array}\right\|}_{2}^{2}+\frac{2\left(N^{\prime }+M^{\prime }\right)}{K+1}$$ (5)

where ${\hat{\theta }}_{i}^{\left(h\right)}$ is the estimated parameters under the assumption that there are *N*′ interacting host proteins and *M*′ interacting pathogen proteins. *K*+1 is the size of data used to estimate ${\hat{\theta }}_{i}^{\left(h\right)}$. For each pathogen protein *j* with *N*′ interacting host proteins and *M*′ interacting pathogen proteins, the AIC value of its dynamic interaction model can be calculated as follows:

$$AIC_{j}\left(N^{\prime }+M^{\prime }\right)=\log\frac{1}{K+1}{\left\|\begin{array}{c} p_{j}^{\left(p\right)}-\Phi_{j}^{\left(p\right)}{\hat{\theta }}_{j}^{\left(p\right)} \end{array}\right\|}_{2}^{2}+\frac{2\left(N^{\prime }+M^{\prime }\right)}{K+1}$$ (6)

where ${\hat{\theta }}_{j}^{\left(p\right)}$ is the estimated parameter assuming *N*′ interacting host proteins and *M*′ interacting pathogen proteins. *K*+1 is the size of the data used to estimate ${\hat{\theta }}_{j}^{\left(p\right)}$. The model order (numbers of interactions) with the minimum AIC value is considered as a criterion to delete false-positive interactions in the candidate HP-PPIN. Specifically, insignificant estimated interaction strengths out of model order were considered as false-positive interactions and were deleted from the candidate HP-PPIN to obtain the resulting HP-PPINs. Hence, the resulting dynamic HP-PPINs comprise dynamic HPI models with model orders of the minimum AIC value. Finally, after identifying the parameters in dynamic interaction models for each host and pathogen protein based on the dual transcriptome data from innate and adaptive phases, the identified interaction parameters ($\alpha_{in}^{\left(h\right)}$, $\alpha_{jm}^{\left(p\right)}$, *γ*_*im*_, and *γ*_*jn*_) complete the resulting dynamic innate and adaptive HP-PPIN (Fig 1C).

## Results

### Overview of dynamic innate and adaptive HP-PPINs

In this study, two dynamic PPINs are constructed for the biologically related conditions: innate and adaptive phases. Dual transcriptome data from host and pathogen in each phase were used to estimate interaction strengths between proteins in dynamic innate and adaptive HP-PPINs. In these analyses, the magnitudes of edges in constructed networks (interaction strengths) were used as quantitative estimates of the effects of one protein on its interacting proteins, implying that the constructed networks are dynamic systems.

**Fig 2:**
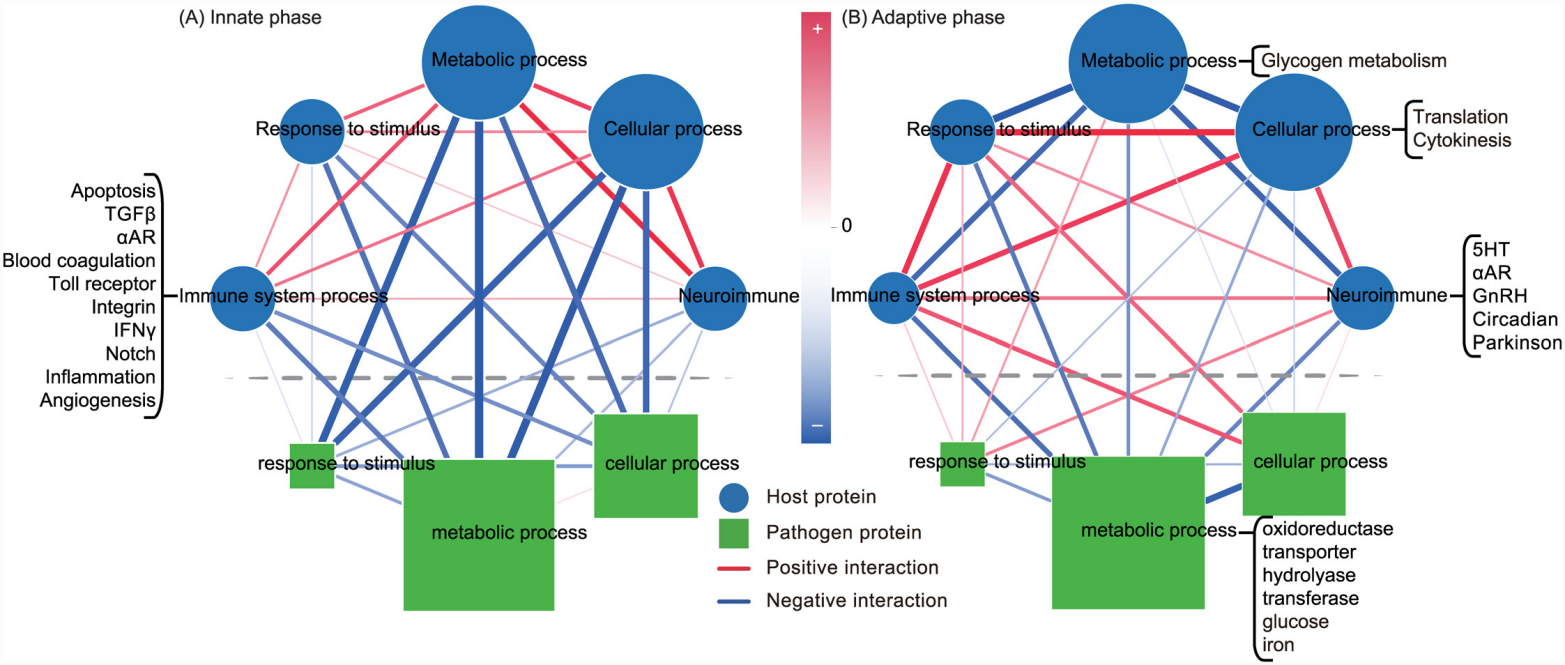
Networks of biological processes enriched in innate and adaptive phases. Innate and adaptive phase-specific networks of enriched biological processes were generated from dynamic innate and adaptive HP-PPINs (Fig 1C), respectively. Node sizes of biological processes indicate numbers of included proteins. Blue and green nodes represent host and pathogen biological processes, respectively, and red and blue edges represent positive and negative interaction strengths between corresponding connecting processes, respectively. Darker edges indicate larger absolute interaction strengths between two biological processes. The functions in open brackets are subjects of subsequent analyses.

Table 1 summarizes the basic information for these dynamic networks and includes the numbers of nodes and edges in dynamic innate and adaptive HP-PPINs (Fig 1C). Although most of host and pathogen proteins are common to innate and adaptive phases, whereas their interactions differ significantly between the two phases. Hence, both host and pathogen use differing interactions between similar sets of proteins to respond to challenges during innate and adaptive phases. Thus, we performed GO annotation and functional enrichment analyses of the proteins in the constructed networks (Fig 1D) to identify the main biological processes involved in the innate and adaptive phases (Fig 2). During the innate phase, all interspecific interactions were negative, indicating that the host and pathogen inhibit each other. In contrast, some positive interspecific interactions were identified in the adaptive phase, indicating enhanced host offenses that are specific to *C. albicans*. In addition, some atypical functions relating to immunity were indicated in the host, namely gonadotropin-releasing hormone receptor pathway, Parkinson’s disease, and circadian clock systems. These neuroimmune functions of the host may be modulated by immunological memory [34–37] in addition to typical host immune-related functions such as inflammation, integrin signaling, and angiogenesis. Therefore, further examination of the networks of biological processes enriched in innate and adaptive phase may indicate the changes required to coordinate host and pathogen molecular mechanisms from innate to adaptive phases.

**Table 1:** Node and edge information of dynamic innate and adaptive host–pathogen protein–protein interaction networks.

Node	Innate-specific	Common	Adaptive-specific
Host	55	856	130
Pathogen	30	1,102	77
Edge	Innate-specific	Common	Adaptive-specific
Host-host	981	633	865
host–pathogen	570	155	374
Pathogen-pathogen	2,356	714	1,664

### Interspecific crosstalk between host immune-related molecular mechanisms and their pathogen counterparts

Hosts can activate and coordinate innate and/or adaptive immune-related components immediately after pathogen invasion according to previous experiences of pathogen exposure. Moreover, both innate and adaptive immune systems serve as defensive mechanisms against pathogen invasion and comprise several coordinated molecular mechanisms including angiogenesis, inflammation, and integrin signaling. However, the coordination of these defensive molecular mechanisms with pathogen counterparts are less addressed. That is, the pathogen functions interacting with host defensive mechanisms and the interaction types are poorly known. Thus, to investigate interactions among immune-related functions and with the pathogen, 120 and 126 host proteins were initially selected from constructed dynamic innate and adaptive HP-PPINs, respectively, based on GO annotations (GO:0002376, immune system process). Subsequently, pathogen counterparts to the immune-related proteins were defined as the ensemble of pathogen proteins with direct connections to immune-related proteins. These host and pathogen proteins and their interactions were further examined in the following analysis. Only 4 and 10 host proteins were specific to the innate and adaptive phases, respectively, and 116 host proteins were common to both. These host immune-related proteins were divided into several functions according to the PANTHER classification system [38] as follows: angiogenesis, inflammation mediated by chemokine and cytokines, notch, interferon-*γ* (IFN-*γ*), intergrin, toll receptor, blood coagulation, *α*-adrenergic receptor (*α*-AR), TGF-*β*, and apoptosis signaling pathways. Moreover, corresponding proteins in the pathogen counterpart were classified into transferase, transporter, oxidoreductase, and hydrolase activities based on GO annotations. Accordingly, these 14 functions (10 in the host and 4 in the pathogen) were organized into subnetworks by summarizing the interaction strengths between members of these functions in the innate and adaptive phases, respectively.

**Fig 3:**
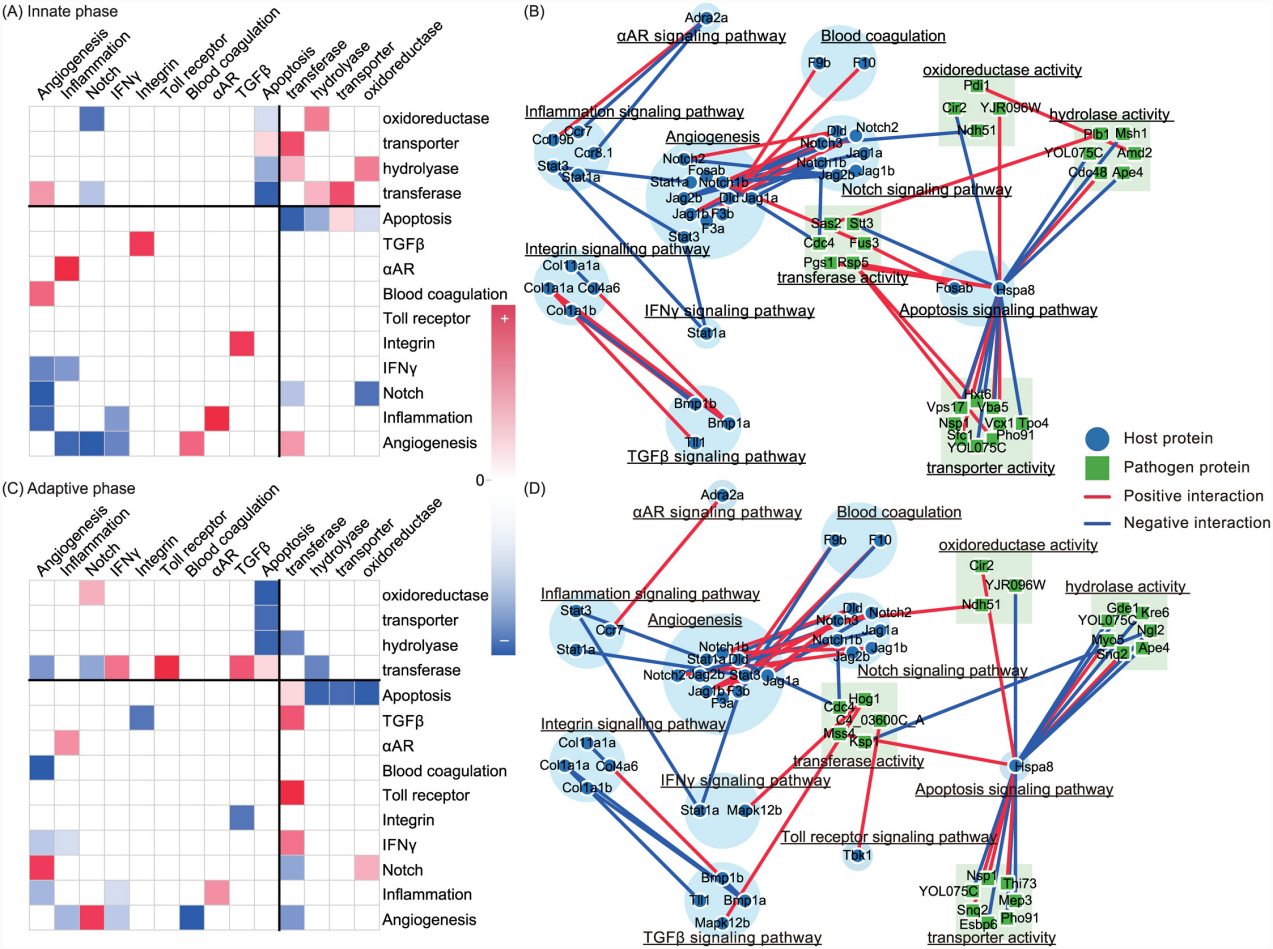
Interspecific crosstalk of host immune-related functions and their pathogen counterparts during innate and adaptive phases at functional and molecular levels. (A and C) denote connectivity between the known pathways of innate and adaptive phases, respectively, and red and blue boxes represent positive and negative interaction strengths, respectively, between the members of two pathways. Darker boxes represent larger absolute interaction strength between two pathways. Names of host and pathogen functions are indicated with upper and lower case letters, respectively. (B and D) denote the subnetworks of host immune-related functions and their pathogen counterparts during innate and adaptive phases, respectively, and blue and green nodes represent host and pathogen proteins, respectively. Red and blue edges represent positive and negative interaction strengths, respectively, between connecting proteins.

Fig 3 shows the connectivity between host immune-related molecular mechanisms and pathogen counterparts during innate and adaptive phases at functional and molecular levels. During innate phase, angiogenesis, apoptosis, and notch signaling pathways were host functions with direct pathogen interactions (Fig 3A). Angiogenesis accessed information of *C. albicans* from multiple growth factor signaling pathways which are responsible for pathogen recognition and initiation of innate immune responses [39]. In particular, Notch1b in angiogenesis and notch signaling pathway interacted negatively with pathogen transferase Cdc4, which is involved in filamentous growth and cell cycle phase transition. Moreover, Fosab in angiogenesis and apoptosis positively interacted with Cek1, which promotes *C. albicans* survival under unfavorable conditions [40]. In the apoptosis hub, Hspa8 had multiple interactions with pathogen proteins, with negative interaction strengths between Hspa8 and pathogen glucose transporters (Hgt6) but positive interaction strengths with pathogen transporters for iron (C1_09210C_A), succinate (Sfc1), H^+^/Ca^2+^ (Vcx1), and PI3P (Vps17; Table 2). The role of the differential interactions between heat shock protein and glucose and other transporters during the innate phase is unclear.

**Table 2:** Downregulated and upregulated interactions between the functions and members of functions in the intraspecific and interspecific crosstalk of host immune-related functions and their pathogen counterparts.

	Downregulated		Upregulated	
Host–host	**Angiogenesis**	**Blood coagulation**	**Angiogenesis**	**Notch**
	F3b	F9b	Jag1b	Notch1b
	F3b	F10	Jag2b	Notch1b
	**Integrin**	**TGF-*β***	Jag2b	Notch3
	Col1a1b	Bmp1a	Jag2b	Notch2
	Col1a1a	Tll1	Notch1b	Jag1a
	Col1a1a	Bmp1a	Jag1b	Notch3
	Col4a6	Bmp1a	Notch1b	Dld
	Col1a1b	Bmp1b	Jag1a	Notch3
	Col11a1a	Bmp1b		
Host–pathogen	**Angiogenesis**	**transferase**	**TGF-*β***	**transferase**
	Fosab	Cek1	Mapk12b	Hog1
	**Apoptosis**	**transporter**	**Notch**	**oxidoreductase**
	Hspa8	Sfc1	Notch3	Ndh51
	Hspa8	Vps17	**Toll receptor**	**transferase**
	Hspa8	Sge11	Tbk1	C4_03600C_A
	Hspa8	Vcx1	**Apoptosis**	**transferase**
	Hspa8	Pho91	Hspa8	Stt3
	Hspa8	C5_03080C_A(ESBP6*)	Hspa8	Mss4
	Hspa8	C1_01920W_A(YOL075C*)		
	Hspa8	Mep1		
Pathogen–pathogen	**oxidoreductase**	**hydrolase**		
	Amd2	Pdi1		
	**transferase**	**transporter**		
	Rsp5	Pho91		
	Rsp5	Hgt6		
	**hydrolase**	**transferase**		
	Sas2	Plb3		
	Ksp1	C6_02580W_A (NGL2*)		

Names of functions are indicated in bold (upper case: host and lower case: pathogen).

* Indicates the yeast ortholog of *Candida albicans* protein.

During the adaptive phase, the numbers of host immune-related functions that interacted with pathogen counterparts were increased in comparison with those during the innate phase (Fig 3C). Moreover, host immune-related functions including IFN-*γ*, TGF-*β*, and toll receptor signaling pathways interacted with the pathogen in addition to angiogenesis, apoptosis, and notch signaling pathways. Mapk12b in IFN-*γ* and toll receptor signaling pathway positively interacted with Hog1, which is a pathogen MAP kinase of osmotic, heavy metal, and core stress responses. Moreover, in the TGF-*β* signaling pathway, TANK-binding kinase 1 (Tbk1) positively interacted with C4_03600C_A, whihc is related to protein sumoylation in yeast and likely inhibits *C. albicans* growth and adaption [41].

In comparisons of the subnetworks shown in Fig 3B and 3D, a repressilator structure among angiogenesis, IFN-*γ*, and inflammation signaling pathways of host emerged in both phases. Because the host can exhibit stable oscillations of immune responses through the repressilator structure, immune responses can be maintained in proportion to the stimulus from the pathogen, thus preserving the continuity of other host functions. Therefore, the repressilator can be viewed as a self-protection mechanism of the host under conditions of no stimulation and no immune responses, and in the presence of pathogens, appropriate immune responses avoid unnecessary damage to host tissues. In addition to the repressilator structure, crosstalk among pathogen functions was greater during the innate phase than adaptive phase. This phenomenon may reflect immunological memory, which drives changes to the coordination of host immune-related functions, enabling the specific inhibition of crosstalk between pathogen functions during the adaptive phase. This immunological memory also enables apoptotic Hspa8 to interact negatively with a broader range of pathogen transporters than the glucose transporter in the innate phase and causes more substantial restriction of nutrient availability and pathogen growth. In addition to transporters, energy metabolism was affected by host immune-related functions, warranting a subsequent focus on resource competition-related molecular mechanisms of pathogens and their host counterparts.

### Interspecific crosstalk of pathogen resource competition-related molecular mechanisms and their host counterparts

Interspecific crosstalk involved 10 and 13 genes related to iron and glucose competition, respectively, which were selected from the constructed dynamic HP-PPINs based on the *Candida* Genome Database (CGD) [42]. The protein products of these 23 genes are involved in iron and glucose utilization in *C. albicans* and are hence referred to as resource competition-related proteins. Host counterparts were defined as the ensemble of host proteins with direct connections to pathogen resource competition-related proteins in innate and adaptive HP-PPINs and were further divided into cytokinesis, translation, circadian, glycogen, and apoptosis functional groups according to GO annotations. The resulting seven functions were then organized into the subnetworks of innate and adaptive phases.

**Fig 4:**
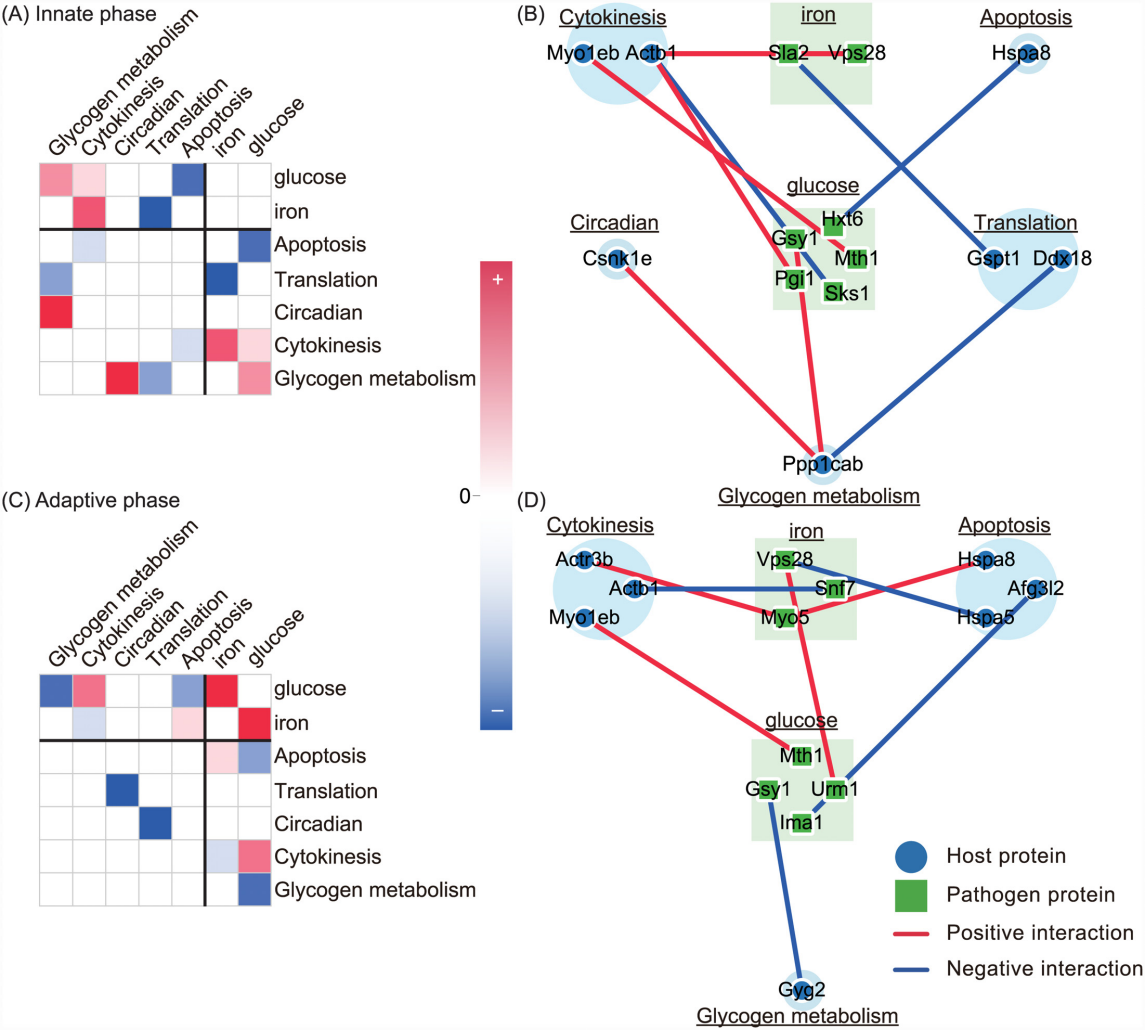
Interspecific crosstalk of pathogen resource competition-related functions and host counterparts during innate and adaptive phases at functional and molecular levels. (A and C) indicate connectivity between known pathways in innate and adaptive phases, respectively, and red and blue boxes represent positive and negative interaction strengths between the members of the pathways, respectively. Darker boxes represent larger absolute interaction strengths between pathways. Names of host and pathogen functions are indicated in upper and lower case letters, respectively. (B and D) indicate the subnetworks of pathogen resource competition-related functions and their host counterparts during innate and adaptive phases, respectively. Blue and green nodes represent host and pathogen proteins, respectively, and red and blue edges represent positive and negative interaction strengths, respectively, between connecting proteins.

Connectivity between pathogen resource competition-related molecular mechanisms and host counterparts in innate and adaptive phases is indicated at functional and molecular levels (Fig 4). During the innate phase, both iron and glucose competition positively interacted with cytokinesis, whereas iron competition negatively interacted with host translation, and glucose competition positively interacted with host glycogen metabolic processes (Fig 4A). Specifically, the endosomal sorting complex Vps28, which is required for the ESCRT-I transport pathway and Pgi1 in iron and glucose competition, positively interacted with the host actin protein Actb1 and promoted host cytokinesis (Table 3), potentially leading to the failure of cytokinesis under the conditions of limited resources [43]. Moreover, among iron competition mechanisms, Sla1 negatively interacted with the host translation termination protein Gspt1, potentially allowing the disruption of the translation termination process and cell cycle arrest [44]. In agreement, Gsy1 reportedly promoted glycogen biosynthesis and affected host translation initiation (Ddx18) through host glycogen metabolic process (Ppp1cab) [45].

**Table 3:** Downregulated and upregulated interactions between the functions and members of two functions in the intraspecific and interspecific crosstalk of pathogen resource competition-related functions and host counterparts.

	Downregulated		Upregulated	
Host–host	**Glycogen**	**Circadian**	**Glycogen**	**Translation**
	Ppp1cab	Csnk1e	Ppp1cab	Ddx18
Host–pathogen	**Cytokinesis**	**iron**	**Cytokinesis**	**glucose**
	Actb1	Vps28	Myo1eb	Std1
	Actb1	Snf7	Actr3b	Myo5
	**Glycogen**	**glucose**	**Apoptosis**	**iron**
	Ppp1cab	Gsy1	Hspa8	Myo5
	Gyg2	Gsy1	**Translation**	**iron**
			Gspt1l	Sla2
Pathogen–pathogen			**iron**	**glucose**
			Vps28	Urm1

Names of functions are indicated in bold (upper case: host and lower case: pathogen).

During the adaptive phase, host translation processes are not regulated by pathogen resource competition-related functions, and whereas the effects of resource competition on host cytokinesis are reduced, host apoptosis signaling is affected by resource competition-related functions (Fig 4C). Snf7 is a component of the endosomal sorting complex that is required for the ESCRT-III transport pathway and negatively interacted with the host actin protein Actb1 to inhibit host cytokinesis. The heat shock proteins Hspa8 and Hspa5 also negatively interacted with pathogen resource competition-related functions, suggesting that the pathogen may abrogate host cell apoptosis to achieve successful invasion during the adaptive phase [46].

In comparison of the two subnetworks of resource competition-related proteins and host counterparts (Fig 4B and 4D), the effects of immunological memory on the coordination of host cytokinesis, translation, and apoptosis were indicated, and interactions with pathogen iron and glucose competition were suggested. During the innate phase, the pathogen promotes host cytokinesis, competes for host resources, and interferes with host translation processes, and the resulting pressure on resource supply weakens host immunity. However, during the adaptive phase, the host has the benefit of immunological memory and avoids resource restriction. Subsequently, pathogen resource competition-related molecular mechanisms engage in intraspecific crosstalk to ensure sufficient resource supply and the pathogen blocks the host apoptosis signaling pathway to avoid attack and achieve successful invasion [3, 46]. These effects of immunological memory on the coordination of defensive and offensive molecular mechanisms in the host and pathogen suggest relationships between immunological memory and the host functions described above.

### Impacts of immunological memory on host systems

To further investigate the impact of immunological memory on host behaviors, host proteins were ranked according to relative interaction strengths in innate and adaptive networks, and significant influences of immunological memory on interaction strengths were indicated. Moreover, this comparative analysis distinguished proteins that are affected by immunological memory, and the top 10-ranked innate- and adaptive-specific host proteins with direct interactions with pathogens were selected. However, because no significant functions were enriched among the selected proteins, the first nearest neighbors were further considered follows. Among innate-specific host proteins, fibroblast, vascular endothelial, and epidermal growth factor signaling pathways were downregulated, indicating positive interaction strengths during the innate phase and zero strength during the adaptive phase. Moreover, apoptosis and circadian clock systems were upregulated, indicating negative interaction strengths during the innate phase and zero strength during the adaptive phase. However, among the adaptive-specific host proteins, the circadian clock system and the pentose phosphate pathway were upregulated (interaction strength, zero in the innate phase and positive in the adaptive phase), and the circadian clock system and the adrenaline biosynthesis were downregulated (interaction strength, zero in the innate phase and negative in the adaptive phase). The pentose phosphate pathway protein Slc18a2 and Synap23.2 are also involved in serotonin (5HT) receptor signaling, which influences circadian rhythms.

In addition to innate- and adaptive-specific host proteins, those involved in common HPIs were further divided into groups of the most and least varied proteins, based on the interaction strengths between innate and adaptive phases. Among most varied proteins, apoptosis and Parkinson’s disease signaling pathways were upregulated, whereas circadian clock and Parkinson’s disease signaling pathways were downregulated. Moreover, among least varied proteins, DNA replication and Parkinson’s disease signaling pathways were upregulated; Parkinson’s disease signaling pathway was downregulated. Accordingly, differing proteins of the Parkinson’s disease signaling pathway is downregulated and upregulated, suggesting tight modulation in both phases [47]. In addition, proteins of *α*-AR signaling, 5HT receptor signaling, and the circadian clock system were closely related. Accordingly, circadian 5HT production is reportedly regulated by adrenergic signaling [48]; 5HT and circadian systems of the brain have been extensively interconnected [49]; and adrenergic nerves were shown to govern circadian leukocyte recruitment to tissues [50]. Hence, circadian clock, Parkinson’s disease, 5HT, and adrenergic signaling pathways are important in HPIs and in defensive and offensive functions of hosts and pathogens.

**Fig 5:**
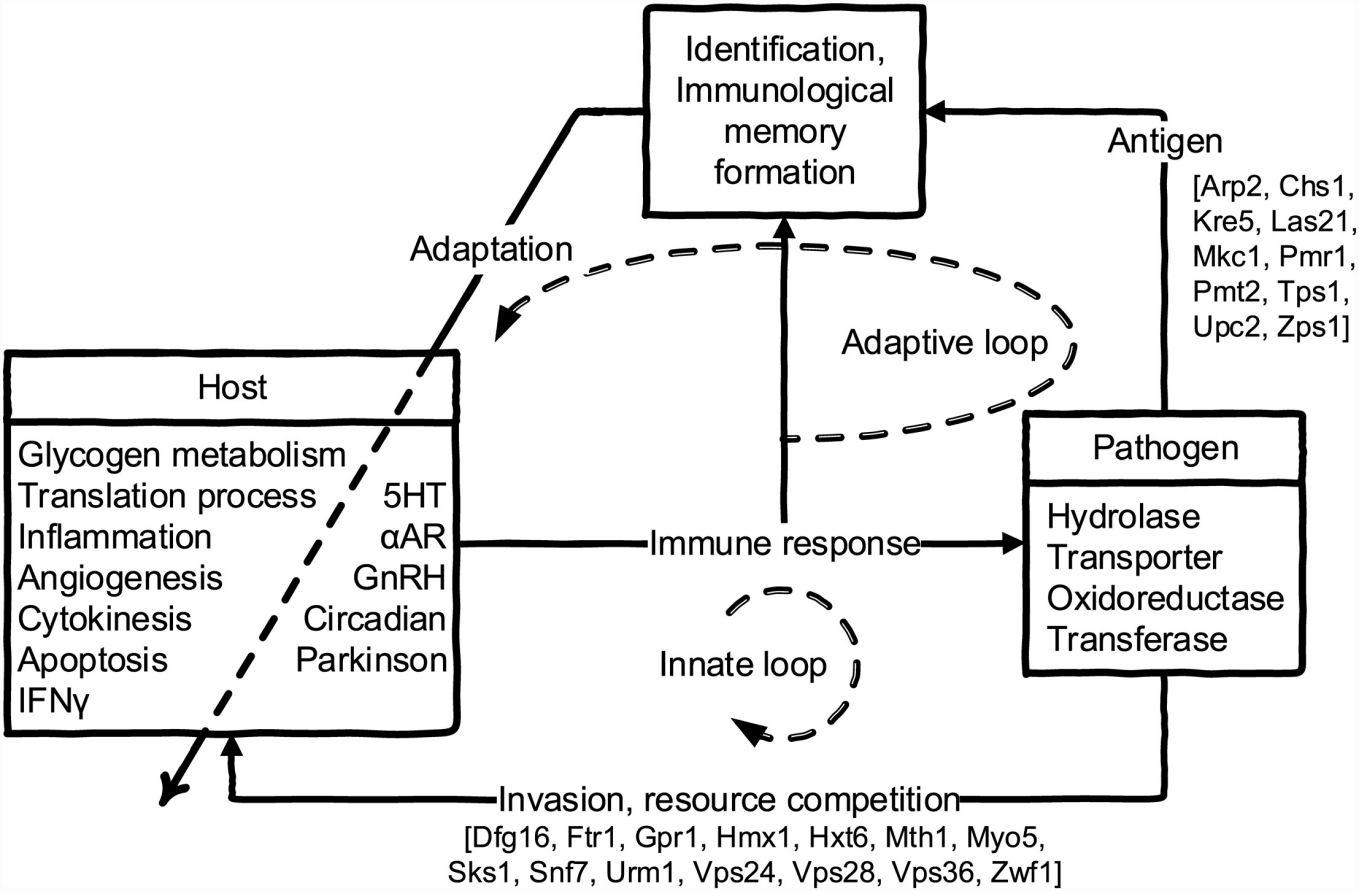
Schematic structure of dynamic host–pathogen interaction (HPI) systems. The dynamic HPI system can be divided into innate and adaptive loops. In the innate loop, the initial invasion leads to antigen presentation to the host, resource competition, and interference with host cellular functions. The host then defends itself using the innate immune response, and pathogenic antigens are identified. In the subsequent adaptive loop, the immunological memory of pathogenic antigens regulates the coordination of host cellular functions based on the identified antigens and host responses. The coordinated functions listed on the left and right hand sides of the dashed line are typical and atypical to immunity, respectively, reflecting systematic immune organization.

Under conditions of poor adaptation of the host system to a specific pathogen, the host coordinates its molecular responses in the innate loop and forms immunological memory of the pathogen (Fig 5) according to the host system responses and pathogen characteristics during the entry into the adaptive loop. Hence, the coordination of molecular mechanisms (the functions related to the host block in Fig 5) is informed by immunological memory, which operates via an adaptive feedback loop to modulate interactions between host molecular mechanisms. This regulation enables the host to identify and adapt to pathogen stimuli and furnishes leukocytes with a repertoire of specific antibodies. As in adaptive control systems, a feedback loop is usually used to achieve adaptation when controlled system dynamics and external disturbances are unknown. Moreover, in analogy to the adaptive control system, immunological memory represents an adaptive feedback mechanism of the host system that modulates host molecular mechanisms by regulating interaction strengths between host and pathogen proteins. In subsequent pathogen challenges, those modulated host functions then exhibit more specific and efficient responses against the pathogen, and enhanced intraspecific pathogen crosstalk disrupts host apoptosis to evade host responses. Hence, both defensive and offensive functions of the host and pathogen and neuroimmune functions are modulated by immunological memory. These neuroimmune functions suggest that nervous and endocrine systems are also coordinated by immunological memory, and the ensuing host defensive and offensive molecular mechanisms are coordinated accordingly.

## Discussion

The present HP-PPINs reflect the coordination of host and pathogen defensive and offensive molecular mechanisms during innate and adaptive phases and suggest new directions for HPI studies. Specifically, subnetworks of host immune-related functions and their pathogen counterparts indicate the presence of a repressilator structure comprising angiogenesis, IFN-*γ*, and inflammation signaling and suggest potential strategies for resolving inflammation. Once pathogen invasion is detected, the repressilator structure can initiate and modulate durations and magnitudes of inflammatory responses according to interspecific crosstalk with pathogen transferases, and host notch, blood coagulation, and *α*-AR signaling pathways. Therefore, the host can actively regulate the resolution of inflammation, even under the conditions of persistent pathogen stimuli [51]. The apoptosis of activated inflammatory cells is key to the resolution of inflammation and was a hub in the present subnetworks (Fig 3). Accordingly, apoptotic heat shock proteins play important roles in interspecific crosstalk with pathogen resource transporters. Moreover, the comparisons of innate and adaptive HP-PPINs indicated differential interactions between heat shock proteins and pathogen glucose transporters and iron, succinate, PI3P transporters, reflecting the regulation of immunological memory and its effects on strategies for resource acquisition and utilization. These observations also indicate how pathogen resource competition-related functions affect host physiology [52].

In contrast with the molecular mechanisms that are regulated by immunological memory, the complement system, a part of the innate immune system, is regarded as a component that does not adapt. In agreement, the comparative analyses of innate and adaptive HP-PPINs showed that the complement system was relatively invariant and was therefore omitted from the networks shown in Fig 3. However, a closer examination of innate and adaptive HP-PPINs (Fig 1C) showed interactions of the complement system with various signaling pathways, including inflammation, plasminogen activating, EGFR, and integrin signaling pathways. Hence, the complement system may predominantly interact with host proteins, because with the exception Pkc1 in *C. albicans* the first and second nearest neighbors of the complement system in both innate and adaptive HP-PPINs were all host proteins. However, the concept of “trained immunity” or “innate immune memory” has been proposed previously [53], warranting further assessment of the invariance of the complement system in innate and adaptive phases.

The subnetwork of pathogen resource competition-related functions and their host counterparts revealed involvements of host translation, cytokinesis, and glycogen metabolisms in the ensuing interspecific crosstalk. During the innate phase, the pathogen restricted resource supply to the host by activating cytoskeleton synthesis and thus promoting host glycogenesis and cytokinesis. This coordination may weaken host immunity. However, during the adaptive phase, the pathogen responds to increased host immune activity by enhancing crosstalk between iron and glucose competition mechanisms and by inhibiting apoptosis. These processes are likely characteristic of the changes in pathogen offensive strategies from innate to adaptive phases. In addition to iron, various micronutrients and trace elements were recently shown to be involved in the regulation of virulence and transcription in *C. albicans*, such as copper, zinc, and magnesium. However, insufficient function annotations were available for copper (0), zinc (3), and magnesium (0), compared with those for iron (70) in the CGD, warranting further studies of these micornutrients. In addition, competition with the host endogenous microbiome requires examining using the systems biology approach.

The present innate and adaptive HP-PPINs indicated the effects of immunological memory on interspecific and intraspecific crosstalk. Specifically, during the innate phase, the host adapts specifically to the pathogen through antigen presentation on dendritic cells and antibody selection in leukocytes. This immunological memory allows more powerful and effective responses during subsequent exposures to the same pathogen, leading to incremental increases numbers of HPIs and decreased intraspecific crosstalk between pathogen functions. Accordingly, various novel predictions are implied by previously unrecognized crosstalk between known pathways, and systematic analyses of the host proteins involved comprise a new research direction [54, 55]. Although the proteins in the most and least varied groups were common to both dynamic innate and adaptive HP-PPINs, differences in interaction strengths are suggestive of the roles of proteins in HPIs. Specifically, proteins in the least varied group were involved in the core conserved molecular mechanisms of innate and adaptive phases, whereas those with large changes (from positive to negative interaction strengths or vice versa) provide more specific and effective responses against the pathogen in accordance with immunological memory. Moreover, neuroimmune functions such as circadian clock, Parkinson’s disease, 5HT, and adrenergic signaling pathways were related to interspecific crosstalk and can affect the infection process and be regulated during adaptation of the pathogen and evolution of immunological memory. However, in the present constructed networks, many genes lacked specific functional annotations, thus limiting the present interpretations. Hence, more evidence for functional annotations may lead to the identification of new functions that have potential to affect host immunity and will validate the present connections between hosts and pathogens.

In summary, Fig 5 depicted the schematic structure of dynamic HPI systems based on the observations at functional and molecular levels. It emphasized the dynamic system viewpoint on the HPI systems and integrated the functions used by host and pathogen to interact each other during the innate and adaptive phases into a self-tuning control system consisted of innate and adaptive loops. During the innate phase, pathogen invasions are inputs to drive the self-tuning HPI systems. The invasion and resource competition activate typical and atypical host functions. In turn, these typical and atypical functions respond to pathogen which completes the innate loop. Immunological memory of pathogen forms based on the host responses and pathogenic antigens and changes the interactions between host proteins and the coordination of the host functions are also changed after innate phase. During the adaptive phase, the challenges of pathogen activate both innate and adaptive loops. The changed coordination of host functions exert specific effects on pathogen which are observed in the comparison of innate and adaptive networks. Thus, the host systems can compute the characteristics based on its responses and pathogen inputs and tune itself through immunological memory. In addition, to identify the coordination of typical molecular mechanisms that are subject to immunological memory, several neuroimmune-related functions have become putative targets of immunological memory. Hence, the present analyses expand the influence of immunological memory and form the basis for new directions in vaccine designs. Further studies of these identified cellular functions and proteins may facilitate translation of host–pathogen relationships to biomedical applications [56].

## Supporting Information

S1 FigThe residual sum of squares and goodness of fit.(A) The distribution of residual sum of squares (RSS) ${\left\|p_{i}-\Phi_{i}\theta_{i}\right\|}_{2}^{2}$ and log of residual sum of squares. (B and C) The comparisons between measured and estimated expression profiles of top 3 largest residual sum of squares during the innate and adaptive phases, respectively.(PDF)
