# Supplementary material for: A Systems Biology Approach to the Coordination of Defensive and Offensive Molecular Mechanisms in the Innate and Adaptive Host–Pathogen Interaction Networks
Source: PLoS One. 2016 Feb 16;11(2):e0149303. doi: 10.1371/journal.pone.0149303 (PMC4755559; doi:10.1371/journal.pone.0149303)

**S1 Figure. The residual sum of squares and goodness of fit.**

(A) The distribution of residual sum of squares (RSS) $\left\|\mathbf{p}_j - \Phi_j \hat{\boldsymbol{\theta}}_j\right\|_2^2$ and log of residual sum of squares.

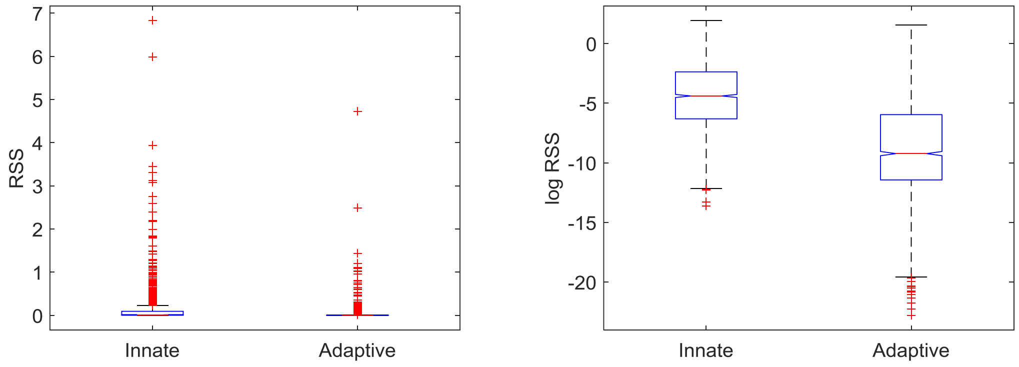


(B) Innate phase

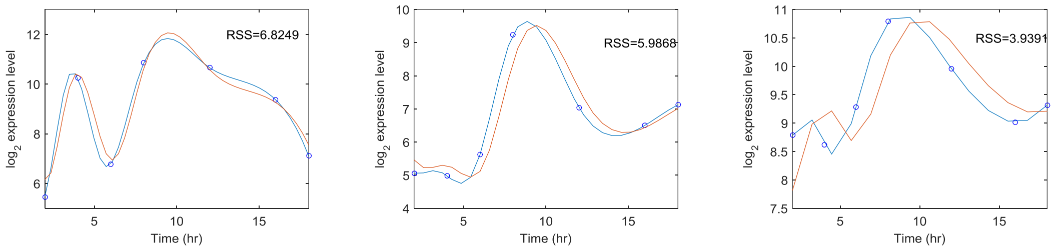


(C) Adaptive phase

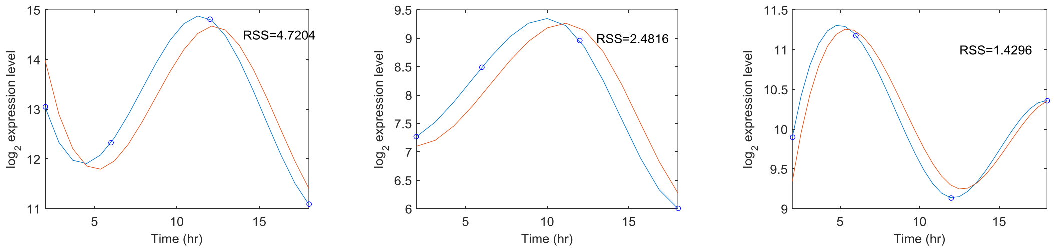

Supplement: S1 Fig — (A) The distribution of residual sum of squares (RSS) ‖pi-Φiθi‖22 and log of residual sum of squares. (B and C) The comparisons between measured and estimated expression profiles of top 3 largest residual sum of squares during the innate and adaptive phases, respectively. (PDF) [file pone.0149303.s001.pdf]
